# Bioengineering the neurovascular niche to study the interaction of neural stem cells and endothelial cells

**DOI:** 10.1063/5.0027211

**Published:** 2021-03-03

**Authors:** Max A. Winkelman, Abigail N. Koppes, Ryan A. Koppes, Guohao Dai

**Affiliations:** 1 Department of Bioengineering, Northeastern University, Boston, Massachusetts 02115, USA; 2 Department of Chemical Engineering, Northeastern University, Boston, Massachusetts 02115, USA

## Abstract

The ability of mammalian neural stem cells (NSCs) to self-renew and differentiate throughout adulthood has made them ideal to study neurogenesis and attractive candidates for neurodegenerative disease therapies. In the adult mammalian brain, NSCs are maintained in the neurovascular niche (NVN) where they are found near the specialized blood vessels, suggesting that brain endothelial cells (BECs) are prominent orchestrators of NSC fate. However, most of the current knowledge of the mammalian NVN has been deduced from nonhuman studies. To circumvent the challenges of *in vivo* studies, *in vitro* models have been developed to better understand the reciprocal cellular mechanisms of human NSCs and BECs. This review will cover the current understanding of mammalian NVN biology, the effects of endothelial cell-derived signals on NSC fate, and the *in vitro* models developed to study the interactions between NSCs and BECs.

## I. INTRODUCTION

It was previously believed that mammalian neurogenesis occurred exclusively during embryonic development. However, the discovery of dividing cells in the adult rat hippocampus challenged that notion.^1^ Later, cells removed from the adult mouse striatum were observed to proliferate and differentiate into neurons and astrocytes *ex vivo.*^2^ Now, it is accepted that adult mammals possess multipotent neural stem cells (NSCs) that maintain neurogenesis throughout adulthood.^3^ Through intrinsic and extrinsic cues, mammalian NSCs maintain their population through a type of asymmetric division called self-renewal, producing two daughter NSCs. NSCs can differentiate into several central nervous system (CNS) cells, including neurons, astrocytes, and oligodendrocytes.^3^ NSCs can also generate neural progenitor cells (NPCs) which share many stem cell attributes but are typically characterized by limited self-renewal and differentiation capacity compared to bona fide NSCs. Given their similarities and lack of established definitions, populations of NSCs and NPCs (NSCs/NPCs) are often described and studied together.^4,5^ The innate abilities of NSCs/NPCs have led to scientific investigations to elucidate the underlying cellular mechanisms that govern their cell fate. This is of particular clinical relevance to cell-replacement therapies for neurodegenerative diseases to replace dead or damaged neural cells.^6,7^ Understanding how NSCs behave in the adult mammalian brain will accelerate the successful clinical application of NSCs in patients with neurodegenerative diseases.

In the adult mammalian brain, NSCs/NPCs are found in two distinct regions: the subventricular zone (SVZ) of the lateral ventricle^8–10^ and the subgranular zone (SGZ) of the dentate gyrus in the hippocampus.^11–13^ In both germinal zones, temporospatial signals in the form of cell–cell contact, soluble growth factors, and extracellular matrix (ECM) proteins regulate NSC self-renewal, quiescence, proliferation, and differentiation.^8,9,11,12^ It was discovered that the majority of proliferating NSCs/NPCs were observed near specialized blood vessels, suggesting the prominent role of endothelial cells in regulating NSC/NPC behavior.^8,14,15^ As a result, the microenvironment in which NSCs/NPCs are maintained is referred to as the neurovascular niche (NVN).^8,16^ Understanding the function of endothelial cells in the NVN will be necessary to elucidate the underlying mechanisms that govern NSC fate.

The successful expansion and differentiation of NSCs *in vitro* has led to enormous progress in basic and applied neurobiology.^17,18^ However, understanding the interaction between endothelial cells and NSCs/NPCs will be imperative for the optimal expansion and differentiation of NSC populations and potential clinical translations.^19,20^ In this review, we will illustrate the current understanding of mammalian NVN biology, identify the vascular contributions that govern NSC/NPC behavior, and evaluate the past and current *in vitro* systems developed to recapitulate and study these cellular interactions.

## II. THE NEUROVASCULAR NICHE

As previously stated, neurogenesis occurs in the SVZ and the SGZ of the adult mammalian brain.^8–13^ Although the cytoarchitecture of these regions differs, both germinal zones contain self-renewing NSCs that give rise to proliferative NPCs which can further differentiate into neuronal and glial cells. Another conserved characteristic is the presence of specialized vasculature that regulates the behavior of the surrounding NSCs/NPCs.^8,14^ This section will describe the structure of both germinal regions and summarize the lineage of the resident stem cells. Due to accessibility and ethics, most physiological data described here will be derived from animal, specifically rodent, models.

### A. Brain blood vessels

The distinctive requirements of the adult mammalian brain constitute the development of unique vascular structures. In the CNS, brain endothelial cells (BECs) form the highly restrictive, semi-permeable blood–brain barrier (BBB) which separates circulating blood from the brain extracellular space. BECs have upregulated expression of tight junction proteins (claudin-5, occludin, and ZO-1), membrane transporters (GLUT-1), and basal lamina proteins (laminin and collagen IV) that contribute to both a physical and transport barrier.^21^ The function of brain microvascular networks is dependent on the synergistic interaction between BECs, pericytes, and astrocytes. Brain pericytes are mural cells that adhere directly to capillaries and regulate microvessel permeability, vasoconstriction, and BBB-specific gene expression.^22^ Astrocytes are glial cells of the CNS that are commonly identified via expression of glial fibrillary acidic protein (GFAP), and can completely ensheath brain blood vessels with perivascular endfeet.^23^ Astroglia upregulate tight junction proteins and polarize BEC membrane protein expression.^23^ Brain blood vessel function is paramount for maintaining homeostasis in the neurovascular unit as well as the NVN.^24^ In the SVZ and SGZ, specialized microvascular networks exhibit sparse astrocyte and pericyte coverage, facilitating direct contact between NSCs/NPCs and BECs.^8,25^ Understanding the significance of these specialized microvessels will be necessary to discover the cellular mechanisms of the NVN.

### B. The neurovascular niche of the subventricular zone

**FIG. 1.**
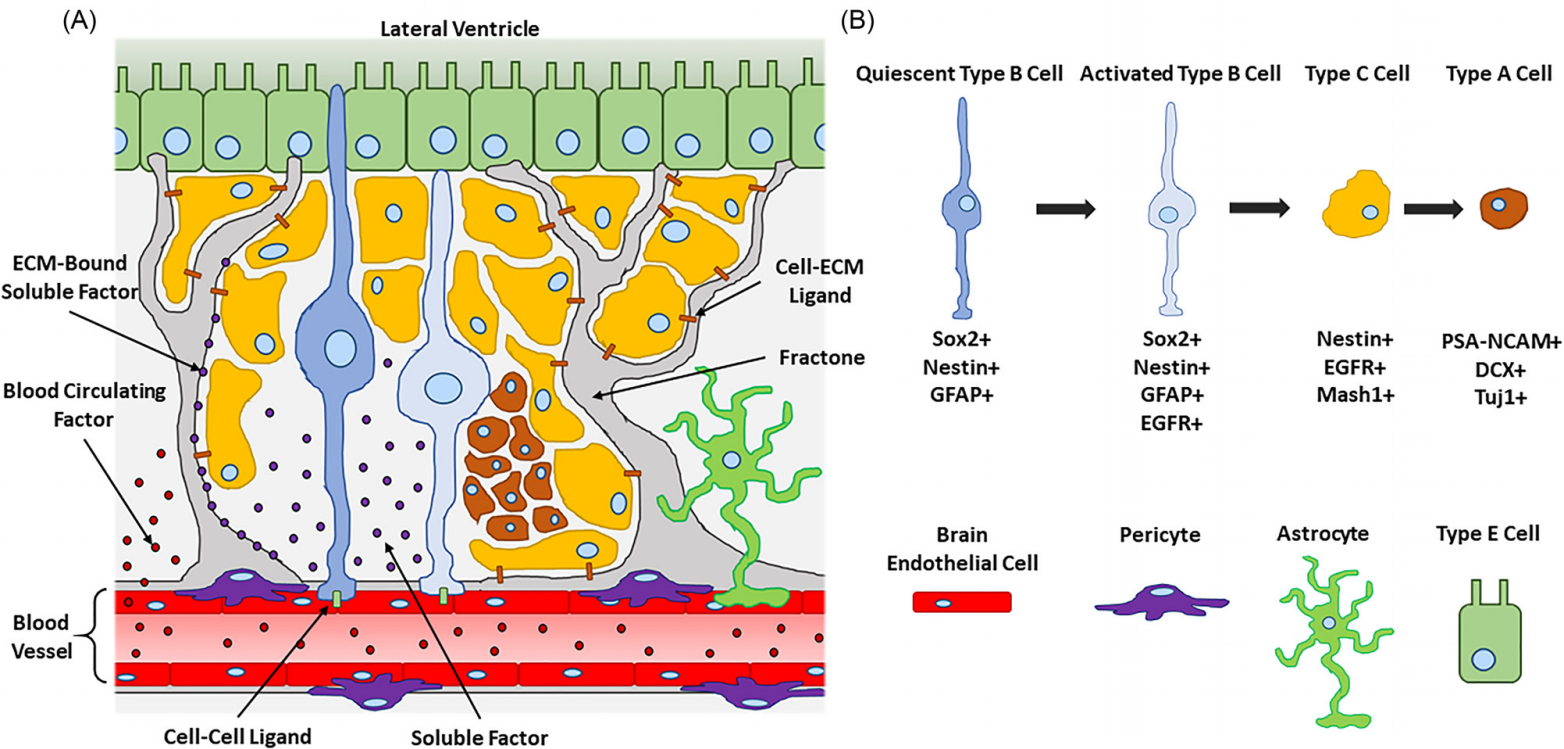
(a) Illustration of the adult rodent neurovascular niche in the subventricular zone (SVZ) of the lateral ventricle. Neural stem cells (NSCs, Type B cells) are found in close proximity to brain blood vessels composed of brain endothelial cells (BECs, red cells), pericytes (purple cells), and astrocytes (green cells). BECs govern NSC behavior through cell–cell (green rectangles) and cell–ECM (brown rectangles) ligands, as well as soluble (purple circles) and blood circulating factors (red circles). Extravascular ECM structures, called fractones (gray structures), capture and activate diffusible signals to enhance their bioactivity. Quiescent Type B cells (blue cells) directly contact endothelial and ependymal cells (Type E cells, rectangular green cells). Activated Type B cells (light blue cells) generate proliferative Type C cells (yellow cells). Type C cells eventually differentiate into neuroblasts (Type A cells, brown cells) which migrate to the olfactory bulb where they become mature neurons. (b) Illustration key with the SVZ NSC lineage diagram showing commonly expressed cell markers. Illustrations not drawn to scale.

The SVZ has been described as a site of neurogenesis in several mammalian species, including humans.^9,26–28^ However, a majority of our mechanistic understanding comes from *in vivo* studies performed in rodents due to their human genome similarities, relatively low cost of care, and capacity for genetic manipulation.^29^ In the rodent SVZ (Fig. 1), near the wall of the lateral ventricle, a unique population of self-renewing, slow-dividing cells called Type B cells were classified as NSCs.^10^ Type B cells extend cellular processes that contact exposed regions of nearby brain capillaries and the ventricular wall which is composed of ependymal cells (Type E cells).^30^ This morphology suggests that Type B cells receive direct contact stimuli from both endothelial and ependymal cells. Due to their astrocyte-like characteristics, Type B cells are identified by the expression of GFAP^10,30–34^ and glutamate aspartate transporter (GLAST).^33–35^ Typical markers of Type B cell self-renewal include the transcription factor, sex-determining region Y-box 2 (Sox2),^36,37^ and the intermediate filament protein, nestin.^2,38^

In general, Type B cells act as either quiescent NSCs (qNSCs) or activated NSCs (aNSCs).^33,34^ qNSCs are slow dividing and remain dormant until they transition to aNSCs. aNSCs are more proliferative and have upregulated expression of the epidermal growth factor receptor (EGFR).^34,39^ aNSCs can transition to progenitor cells called Type C cells which lose their GFAP expression^33,34^ and begin to express the neuronal differentiation transcription factor, Mash1.^33,40^ While both Type B and Type C cells are found near blood vessels, Type C cells are the majority of proliferative cells in the SVZ and undergo multiple rounds of mitosis to generate a sizable population before differentiating.^8^ Type C cells committed to becoming neurons give rise to neuroblasts (Type A cells) which contact blood vessels less intimately.^8^ Due to their neuronal lineage, a majority of neuroblasts express Class III β-tubulin (TuJ1)^33,41^ and doublecortin (DCX).^42^ A small population of Type A cells committed to becoming oligodendrocytes express oligodendrocyte lineage transcription factor 2 (Oligo2)^43,44^ and galactocerebroside (GalC).^45^ Nonetheless, all neuroblasts are highly motile and express the polysialylated-neural cell adhesion molecule (PSA-NCAM).^46^ Neuroblasts eventually leave the SVZ and migrate to the olfactory bulb via the rostral migratory stream (RMS).^47,48^ These neuroblasts migrate longitudinally to blood vessels that run parallel to the RMS, suggesting that capillaries act as migratory scaffolds.^47^ Once at the olfactory bulb, neuroblasts become mature interneurons^49,50^ and express microtubule-associated protein 2 (MAP2).^51^ In summary, these observations indicate that blood vessels have an active role in influencing cell behavior throughout the process of neurogenesis in the SVZ.

### C. The neurovascular niche of the subgranular zone

Although less is documented about the mammalian SGZ, it shares several characteristics with the SVZ. Adult hippocampal neurogenesis has been observed in several mammals, including humans.^11,12,14,52^ The long-term potentiation or the high degree of synaptic formation and pruning within the hippocampus is paramount for memory encoding and processing. This physiologic plasticity and neurogenesis are reliant on the immune system and the proximity of vasculature. In the rodent hippocampus, radial glia have been identified as the resident stem cells and are commonly referred to as Type 1 cells.^52^ Like Type B cells, Type 1 cells express astroglia (GFAP)^13,25,53^ and stem cell markers (Nestin and Sox2).^54,55^ Type 1 cells also extend radial processes that contact local blood vessels and maintain their population by rarely dividing.^52,55^ When they do divide, Type 1 cells undergo asymmetric cell division to produce a Type 1 cell and a Type 2 cell.^53^ Type 2 cells are highly proliferative and typically express nestin and DCX.^25,53^ These cells eventually transition to neuroblast-like Type 3 cells (Nestin-, DCX+, PSA-NCAM+).^25,53^ Type 3 cells migrate tangentially along blood vessels before terminating in the granule cell layer where they differentiate into mature dentate granule neurons.^52,54^ Although the majority of Type 2 cells undergo neurogenesis, clonal lineage-tracing revealed that radial glia can differentiate directly into astrocytes, but not oligodendrocytes.^56^ Differences in cytoarchitecture aside, both the mammalian SVZ and SGZ have NSCs whose progeny are regulated by local blood vessels.

## III. VASCULAR CONTRIBUTIONS IN THE NEUROVASCULAR NICHE

Endothelial cells employ multiple mechanisms to influence other cell types: (1) juxtacrine signaling with adjacent cells using cell membrane and ECM proteins; (2) paracrine signaling with proximal cells using diffusible growth factors; and (3) endocrine signaling with distant cells using hormones released into the circulatory system. In this section, we will describe the vascular signals of the NVN and how they influence NSC/NPC self-renewal and differentiation (Table I). Due to the reasons mentioned previously, most of the experimental data discussed in this section will be from rodent *in vivo* studies or utilize rodent-derived cells.^29^

**TABLE I.** Interactions of endothelial cells and neural stem cells.

Molecule	Cell source	Mechanism	Effect	References
Ephrin-B2	Endothelial	Cell–cell contact	• Bonded neural Eph receptor and inhibited NSC proliferation and promoted quiescence	37
Jagged1	Endothelial	Cell–cell contact	• Bonded neural Notch receptor and inhibited NSC proliferation and promoted quiescence	37
MCAM	Endothelial	Cell–cell contact	• Bond neural matriptase (MTP) and induced BEC secretion of cytokines and chemokines	57, 58
BDNF	Endothelial	Soluble factor	• Promoted neuron recruitment and survival through TrkB-dependent mechanism	59–62, 65
			• Reduced NSC/NPC proliferation and increased neuronal differentiation	
			• Promoted neuroblast migration	
NT-3	Endothelial	Soluble factor	• Bonded neural TrkC and enhanced NSC production of NO	66
			• Reduced aNSC proliferation and promoted quiescence	
PEDF	Endothelial	Soluble factor	• Promoted NSC self-renewal	67, 69
			• Induced NSC symmetric cell division through Notch pathway signaling	
SDF1	Endothelial	Soluble factor	• Bonded neural CXCR4	68
			• Promoted migration of EGFR+ aNSCs and NPCs toward blood vessels	
			• Promoted migration of neuroblasts away from the SVZ niche	
MMP2 and MMP9	Endothelial	Soluble factor	• Degraded brain ECM and enhanced NPC and neuroblast migration	70
BTC	Endothelial	Soluble factor	• Bonded neural EGFR and promoted NSC proliferation and stymied spontaneous differentiation	76
PlGF-2	Endothelial	Soluble factor	• Bonded neural VEGF-R1 and enhanced aNSC and NPC proliferation	77
EGFL7	Endothelial	Soluble factor	• Promoted Jagged1-induced Notch signaling and inhibited aNSC proliferation	78
			• Promoted Delta-like ligand 4-induced Notch signaling and induced NSC quiescence	
sAPP	Endothelial	Soluble factor	• Decreased qNSC proliferation	81
BMP2 and BMP4	Endothelial	Soluble factor	• Decreased NSC/NPC proliferation	82
TGF-β	Endothelial	Soluble factor	• Decreased NSC proliferation and induced apoptosis	83
Laminin	Endothelial	Cell–ECM contact	• Promoted NSC/NPC adherence to blood vessels	87, 88
			• Induced NSC activation when depleted	
			• Regulated NPC proliferation and migration via integrin α6β1 binding	
HSPG	Endothelial	ECM-bound soluble factor	• Bonded bFGF and enhanced NSC proliferation	92–94
			• Bonded BMP4 and BMP7 and reduced NSC proliferation	
EPO	Endothelial	Blood circulating factor	• Increased secretion of endothelial MMP2 and MMP9	97, 98
			• Increased NSC/NPC proliferation and neurogenesis	
Prolactin	Endothelial	Blood circulating factor	• Increased NPC proliferation and olfactory neurogenesis	99, 100
Growth Hormone	Endothelial	Blood circulating factor	• Increased NSC proliferation	100, 101
GDF11	Endothelial	Blood circulating factor	• Increased number of Sox2+ NSCs	102
CCL11	Endothelial	Blood circulating factor	• Decreased neurogenesis	103
VEGF	Neural	Soluble factor	• Enhanced blood vessel density	104–106
			• Guided brain angiogenesis	
			• Enhanced microvascular network density after ischemia	
HIF-1α	Neural	Soluble factor	• Enhanced microvascular network density after ischemia	106

### A. Direct endothelial cell contact

Juxtacrine signals are essential for maintaining NVN homeostasis. Indeed, direct cell–cell contact between brain microvessels and Type B cells was hypothesized to be critical in promoting NSC stemness.^8^ Ottone and colleagues observed endothelial ephrin-B2 and Jagged1 binding to neural Eph and Notch receptors, respectively, at locations where NSC projections contacted exposed BECs.^37^ This binding inhibited NSC proliferation and promoted quiescence.^37^ In the mouse SVZ, Type C and Type A cells contacted blood vessels less than Type B cells, reducing their interaction with BEC surface proteins and mitigating the downstream effects.^8^ Cell–cell contact between NSCs and BECs also activates signaling pathways in the latter. Tung and Lee observed that the binding of NSC/NPC transmembrane protease, matriptase (MTP), with BEC melanoma cell adhesion molecule (MCAM) induced endothelial G protein activation.^57^ This further promoted mitogen-activated protein kinase signaling and engendered the secretion of cytokines and chemokines from endothelial cells.^57,58^ These data demonstrate the reciprocal modulation between NSCs and BECs and reveal how direct cell–cell contact maintains a population of NSCs throughout mammalian adulthood.

### B. Endothelial cell-derived soluble factors

In addition to the delivery of nutrients, blood vessels produce and secrete diffusible growth factors that influence neighboring cells. Although there are numerous soluble niche signals that modulate NSC/NPC behavior, the following soluble factors have been postulated to be of endothelial cell origin. A prominent endothelial cell-derived neurotrophin is brain-derived neurotrophic factor (BDNF). The BEC-derived BDNF was observed to stimulate neuronal recruitment in rodent brain explants through a tropomyosin receptor kinase B (TrkB)-dependent mechanism.^59^ Louissaint and colleagues demonstrated that testosterone induced angiogenesis in the adult songbird higher vocal center by upregulating the local expression of the vascular endothelial growth factor (VEGF) and VEGF receptor 2 (VEGF-R2).^60^ The additional blood vessel formation resulted in increased production of vascular BDNF which promoted neuronal recruitment.^60^ These data confirmed that angiogenesis and neurogenesis were coupled processes. It was later discovered that vascular BDNF promoted the NSC/NPC production of nitric oxide (NO) which reduced NPC proliferation and increased neuron differentiation.^61^ Li and colleagues proposed a positive feedback loop in which NSC-derived NO promoted the production of endothelial VEGF and BDNF; The latter of which stimulated additional NSC NO production and subsequent neuogenesis.^62^ Although VEGF is expressed by endothelial cells, sources of VEGF in the brain include astrocytes^63^ and neurons,^64^ making the specific effect of vascular VEGF on NSC/NPC behavior difficult to quantify *in vivo*. Finally, BEC-derived BDNF was found to guide migrating neuroblasts along RMS blood vessels, demonstrating the significance of vascular BDNF signaling at multiple stages of NSC lineage.^65^

Another neurotrophic factor, neurotrophin-3 (NT-3), was found to be secreted by BECs in the mouse SVZ.^66^ The binding of vascular NT-3 to neural TrkC enhanced NSC production of NO which acted as a cytostatic factor for aNSCs and induced quiescence.^66^ In the mouse SVZ, both endothelial and ependymal cells were shown to secrete pigment epithelium-derived factor (PEDF)^67^ and stromal-derived factor 1 (SDF1).^68^ Vascular PEDF stimulated NSC self-renewal, as demonstrated by increased expression of Sox2 and Notch downstream effectors (Hes1 and Hes5).^67^ Through the Notch pathway, vascular PEDF also promoted the symmetric cell division of NSCs.^69^ In the mouse SVZ, soluble SDF1 acts by binding to CXC chemokine receptor 4 (CXCR4) expressed on Type B, Type C, and Type A cells.^68^ Through a CXCR4-dependent mechanism, vascular SDF1 stimulated two forms of chemotaxis: (1) activated Type B cells (GLAST+, EGFR+) and Type C cells (EGFR+) migration toward blood vessels; and (2) Type A cells (PSA-NCAM+) migration out of the SVZ. Furthermore, it was reported vascular SDF1 did not affect quiescent Type B cells (GLAST+, EGFR-, CXCR4-).^68^ This study demonstrated that the migratory effect of vascular-derived diffusible signals can be dependent on the stage of NSC lineage. Wang and colleagues observed that neuroblasts exhibited enhanced migration to sites of cerebral ischemia in mice infused with erythropoietin (EPO).^70^ Mouse BECs exposed to EPO became “activated” and secreted matrix metalloproteinase 2 (MMP2) and MMP9 which promoted the migration of NPCs.^70^ Taken together, these data suggest that endothelial cell-derived MMPs increase brain ECM degradation which facilitates the migration of several SVZ cell types.

NSC/NPC proliferation can be promoted with the epidermal growth factor (EGF) and basic fibroblast growth factor (bFGF) *in vitro.*^71^ While both proteins are secreted by endothelial cells, there are various other cell sources of EGF^72,73^ and bFGF^74,75^ in the mammalian brain and the contributions from endothelial cells are speculative. However, human umbilical vein endothelial cells (HUVECs) were shown to secrete betacellulin (BTC), an EGF family protein, which promoted mouse NSC proliferation and stymied spontaneous differentiation through EGFR binding.^76^ Similarly, Crouch and colleagues identified placental growth factor 2 (PlGF-2), a VEGF family protein, as a mitogen derived from mouse SVZ BECs that had a proliferative effect on aNSCs and NPCs through VEGF-R1 binding.^77^ In contrast, EGF-like domain-containing protein 7 (EGFL7) secreted by mouse endothelial cells reduced aNSC proliferation by promoting Jagged1-induced Notch signaling at the interface between NSCs and blood vessels.^78^ Vascular EGFL7 also induced aNSC quiescence by promoting Delta-like ligand 4-induced Notch signaling, demonstrating two distinct mechanisms through which EGFL7 influenced the Notch signaling pathway.^78^ The proliferative effect of a growth factor can also be dependent on the target cell. As an example, soluble amyloid precursor protein (sAPP) is a cleaved transmembrane protein that promoted the proliferation of aNSCs and NPCs expressing EGFR in the adult rodent SVZ^79^ and SGZ.^80^ However, endothelial cell-derived sAPP decreased qNSC proliferation in the SVZ, suggesting contrasting effects of sAPP on qNSCs and aNSCs.^81^ Moreover, Mathieu and colleagues identified adult mouse BECs as a source of the bone morphogenetic proteins (BMPs), BMP2 and BMP4, which decreased NSC/NPC proliferation *in vitro* even in the presence of EGF and bFGF.^82^ In the SVZ of irradiated mice, increased BEC expression of transforming growth factor beta (TGF-β) was accompanied by decreased NSC proliferation.^83^ Blocking of TGF-β signaling increased NSC proliferation and stymied apoptosis, revealing the neurotoxic effects of vascular signals during brain injury.^83^ In summary, there are numerous paracrine signals from endothelial cells that influence NSC/NPC behavior. However, the cell source and effect of many niche factors remain speculative and require further research to confirm.^16^

### C. Blood vessel extracellular matrix interactions

Brain blood vessels are encompassed by basal lamina composed of collagen IV, laminin, and heparin sulfate proteoglycans (HSPG).^84,85^ Basal lamina contributes to the structure and function of the BBB and has a significant regulatory role in the NVN. Flanagan and colleagues found that human NSCs/NPCs expressed laminin-binding integrin subunits (α3, α6, α7, β1, and β4) and exhibited enhanced migration, proliferation, neurogenesis, and gliogenesis on laminin-coated substrates *in vitro.*^86^ In addition to BECs, Type E, Type B, Type C, and Type A cells all contribute to the laminin content of the mouse NVN microenvironment.^87^ Interestingly, while Type C and Type A cells were shown to express high levels of laminin-binding integrins, quiescent Type B cells did not.^87^ However, when the SVZ population of Type C and Type A cells was reduced, the laminin composition decreased and quiescent Type B cells became activated and upregulated laminin-binding integrins.^87^ These results suggest that aNSCs produce laminin-binding integrins to interact with laminin-coated blood vessels to increase their proliferation and re-populate the SVZ with progenitor cells. Indeed, it was shown that the laminin receptor, integrin α6β1, is required for NPCs to adhere to BECs in the adult mouse SVZ.^88^ Blocking of integrin α6β1 caused NPCs to migrate away from blood vessels and proliferate, further demonstrating the regulatory role of vascular laminin.^88^

In addition to providing cell–ECM binding sites, the basal lamina of blood vessels acts as a net to capture and activate soluble factors in the NVN. In the SVZ and SGZ, blood vessels possess extravascular ECM structures called fractones which are composed of collagen IV, laminin, and HSPGs.^88–91^ It was observed that most mitotic cells in the SVZ were located in near fractones composed of N-sulfate HSPG which captured bFGF.^91^ Douet and colleagues found that the proliferative effect of bFGF on NSCs required HSPG association, suggesting that fractones activate diffusible growth factors.^92^ Similarly, the inhibitory effect of both BMP4^93^ and BMP7^94^ on NSC proliferation was found to be dependent on the binding with HSPGs in fractones. Given the necessity of extravascular ECM structures for proper growth factor function, further attention must be given to blood vessel fractones as regulatory components of the NVN.

### D. Blood circulating factors

The endocrine system has been shown to regulate stem cell niches through the delivery of cytokines and hormones via the circulatory system.^95^ As previously mentioned, EPO-infused mice demonstrated increased secretion of BEC MMPs, which enhanced NPC migration.^70^ Endogenous EPO has a neuroprotective effect and is produced in the brain in a hypoxia-dependent manner.^96^ Infusion of EPO into the adult mouse SVZ was shown to enhance NSC/NPC proliferation and neurogenesis.^97,98^ Similarly, the infusion of prolactin, a pituitary gland maternity hormone, into ovariectomized mice increased SVZ NPC proliferation and enhanced the generation of olfactory interneurons.^99^ Like prolactin, the pituitary gland stress hormone, growth hormone (GH) promoted human fetal NSC (fNSC) proliferation and migration *in vitro.*^100^ Blackmore and colleagues found that increased GH secretion during voluntary exercise increased NSC populations in mice, giving credence to exercise-induced neurogenesis.^101^ Blood circulating effectors have also been hypothesized to influence the age-dependent decrease in neurogenesis. For example, Katsimpardi and colleagues observed that BMP11 (GDF11) was more concentrated in the serum of young mice than old mice.^102^ Daily injections of recombinant GDF11 into old mice enhanced vascular remodeling and increased the number of Sox2+ NSCs.^102^ Conversely, Villeda and colleagues observed higher concentrations of C–C motif chemokine 11 (CCL11) in the blood of old mice.^103^ Increasing peripheral CCL11 levels in young mice decreased neurogenesis and impaired learning and memory.^103^ Taken together, these data demonstrate how understanding the mechanisms of blood-borne factors in the NVN will be critical for the development of future CNS therapeutics that rely on delivery through the circulatory system.

### E. Neural contributions in the neurovascular niche

Since blood vessels act as the major regulators in the mammalian NVN, there is significantly more information about the effects of BECs on NSCs/NPCs than the reciprocal influence. However, *in vivo* models have identified blood vessel responses to neural stimuli. Haigh and colleagues discovered that reduction of VEGF-A expression in NPCs decreased blood vessel density and branching in the developing CNS of embryonic mice and eventually led to hypoxia and neuronal apoptosis.^104^ A similar study demonstrated that secretion of VEGF from NPCs guided brain capillary angiogenesis toward the mouse neonatal ventricular zone.^105^ These data indicate that NPC paracrine signals are critical for proper vascular network formation in early mammalian development. Furthermore, NSC/NPC-derived hypoxia-inducible factor 1α (HIF-1α) and VEGF promoted brain microvascular network stabilization after ischemia was induced in young mice, suggesting a therapeutic application of NSCs/NPCs could promote vasculogenesis after adult brain injury.^106^ Finally, Lacer and colleagues observed an increase in the percentage of S phase Sox2+ NPCs in the postnatal mouse SVZ after the ventricular injection of EGF and bFGF. This increase in mitotic NPCs was accompanied by enhanced blood flow to the SVZ, demonstrating neurometabolic coupling in the NVN.^107^ Although the sources presented have identified that NSCs/NPCs do indeed influence blood vessel formation and function, only Lacer and colleagues explored the effect on blood vessels in the adult NVN. Moreover, the mechanism responsible for the neurometabolic coupling was not identified. The implementation of *in vitro* NVN models could reveal the reciprocal coupling between BECs and NSCs/NPCs at the cellular level.

## IV. MODELING THE INTERACTION BETWEEN NEURAL STEM CELLS AND ENDOTHELIAL CELLS *IN VITRO*

**FIG. 2.**
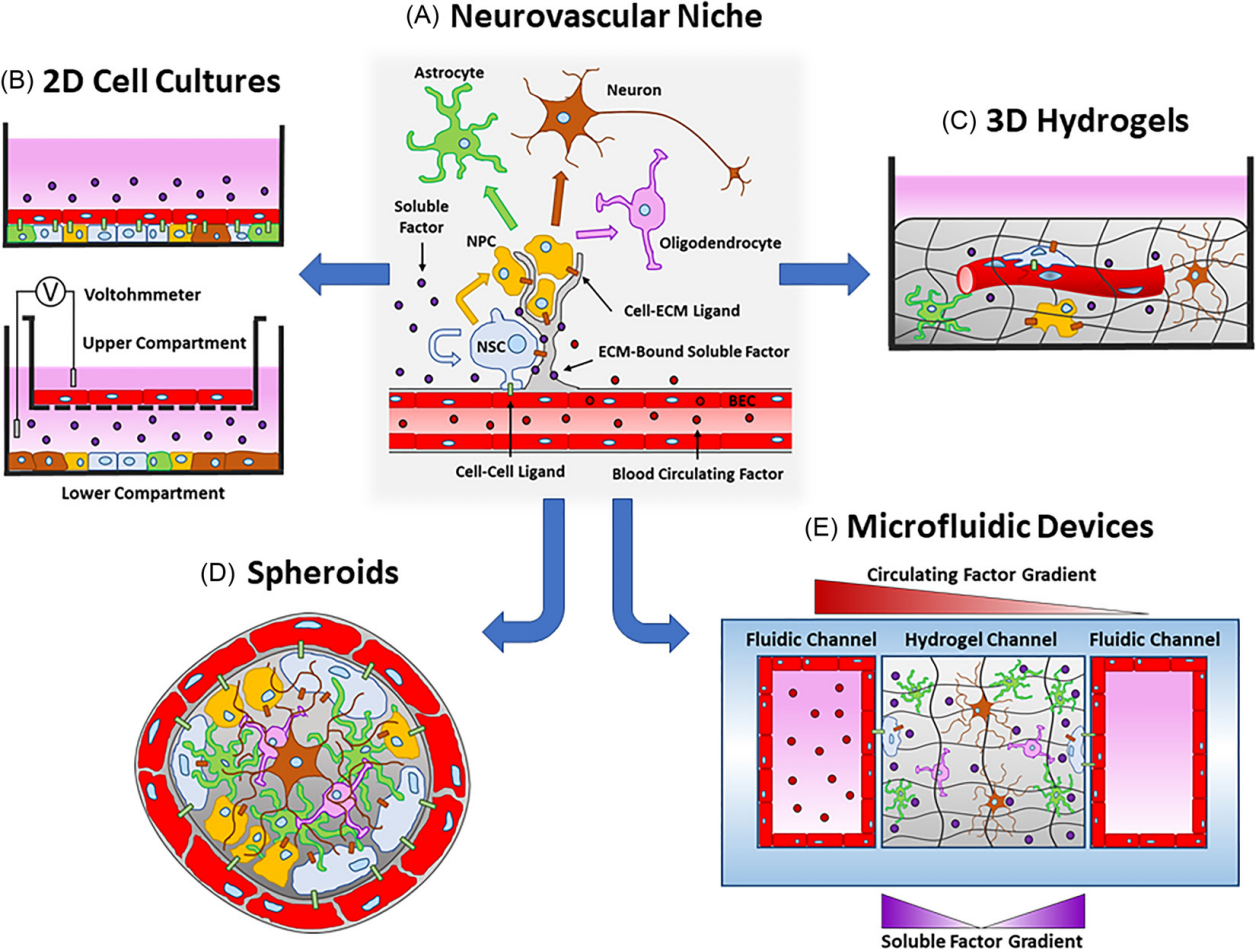
Illustration of the systems used to model the interactions between brain endothelial cells (BECs, red cells) and neural stem cells (NSCs, blue cells). (a) The neurovascular niche (NVN) contains self-renewing NSCs that generate proliferative neural progenitor cells (NPCs, yellow cells) which can differentiate into neurons (brown cells), astrocytes (green cells), and oligodendrocytes (pink cells). BECs govern NSC and NPC behavior through cell-cell (green rectangles) and cell-ECM (brown rectangles) ligands, as well as soluble (purple circles) and blood circulating factors (red circles). The *in vitro* models created to study the NVN include 2D cell cultures (b), 3D hydrogels (c), spheroids (d), and microfluidic devices (e). Illustrations not drawn to scale.

Understanding the reciprocal interactions between endothelial cells and NSCs/NPCs will be critical to advance their clinical application in humans. However, most of the data about the vascular and neural contributions in the NVN have been procured from rodent studies. Animal studies, particularly with mice, act as CNS models physiologically similar to humans with the added feature of genetic modification.^29^ While animal models are biologically complex, experimental results suffer from problems with cross-species translatability that lead to low animal-to-human predictability, especially in clinical drug development.^108,109^ Despite the genetic similarities between mice and humans, their genomes are not identical. It is generally agreed upon that the increased utilization of human cell types will lead to higher success rates in clinical trials. In addition, the shift away from animal-based studies mitigates the ethical concerns raised when using animal models. However, the physical and ethical limitations of using human subjects prevent the comprehensive investigation needed to identify cellular and molecular interactions in the native human NVN. For these reasons, the development of *in vitro* systems has accelerated to produce predictive models of the human biology. As previously stated, the origin and effect of many niche factors remain speculative and require further confirmation. Furthermore, the discovery of niche factors in rodent models does not guarantee the similar presence or consequences in humans. Therefore, the development of complex *in vitro* models is needed to elucidate the cellular interactions between endothelial cells and NSCs/NPCs in humans. In this section, we will discuss two-dimensional (2D) cell cultures [Table II, Fig. 2(b)], three-dimensional (3D) hydrogels [Table III, Fig. 2(c)], spheroids [Table IV, Fig. 2(d)], and microfluidic devices [MFDs, Table V, Fig. 2(e)], as well as the relative advantages and disadvantages of each. While the development of accurate *in vitro* models of the human NVN is a long-term goal for the field, most of the 2D cell culture models that will be discussed used rodent cells due to their ease of acquirement and acceptance at the time of the study. Finally, an in depth discussion of *in vitro* systems using other NVN cell types will be minimized to highlight the contribution from endothelial cells.

**TABLE II.** Two-dimensional cell cultures.

Experimental design	Major findings	References
NSCs/NPCs isolated from adult mouse SVZ or NSCs/NPCs differentiated from mouse 46C ESCs were co-cultured directly on top of bEnd.3 cells in 100-mm^2^ cell culture dishes	• Binding of NSC/NPC MTP with BEC MCAM induced secretion of endothelial cytokines and chemokines	57, 58
NSCs isolated from adult mouse SVZ were co-cultured directly on top of bEnd.3 cells or primary mouse brain microvascular endothelial cells in cell culture dishes	• BECs induced NSC quiescence through the binding of endothelial ephrin-B2 and Jagged1 to neural Eph and Notch receptors, respectively	37
fNSCs derived from the ganglionic eminence or the cortical anlage were cultured on a monolayer of hCMEC/D3 cells in 24-well plates	• BECs cultured on fNSCs formed vascular-like structures with tight junction and basement membrane proteins	111, 112
	• Ganglionic eminence fNSCs induced BEC tube formation four times as efficiently as cortical anlage fNSCs	
	• fNSC neurogenesis was significantly increased in the presence of vascular-like structures	
	• Tubular network formation was mediated by VEGF-R2 and laminin-binding integrin subunit α6	
Mouse NPCs (NE-4C) were co-cultured with mouse embryonic PVECs, adult BECs, and adult dermal microvascular endothelial cells in 96-well Matrigel-coated plates	• All endothelial cells formed tubular networks	121
	• PVECs exhibited increased tube length when co-cultured with NPCs	
Mouse embryonic (ACTβEGFP) NSCs/NPCs were co-cultured on top of bEnd.3 cells in Matrigel-coated 12 mm diameter coverslips or indirectly co-cultured in Transwells. Cell cultures were subjected to OGD injury.	• HIF-1α and VEGF expression was upregulated in NSCs/NPCs co-cultured with BECs under OGD conditions	106
	• BEC morphogenesis was induced through neural HIF-1α/VEGF-related mechanisms	
Fluorescence activated cell sorting (FACS)-purified qNSCs, aNSCs, and NPCs from 2-month-old GFAP-GFP mice were cultured in media conditioned with FACS-purified endothelial cells and pericytes from the SVZ or the cortex of CD-1 2-month-old male mice	• Soluble factors from endothelial cells and pericytes from both brain regions promoted NSC/NPC proliferation and neurogenesis	77
	• Diffusible signals from cortical vascular cells had the most the influential effect	
NPCs isolated from the SVZ of adult Swiss Webster mice were cultured in media conditioned with bEnd.3 cells exposed to static or flow conditions using a cone-plate viscometer	• Conditioned medium from static and flow conditions promoted oligodendrocyte and neuron differentiation, respectively	126
	• BECs demonstrated enhanced expression of EGF, bFGF, and HSPGs in the flow condition	
Primary bovine pulmonary artery endothelial cells, bEnd.3 cells, vascular smooth muscle cells, NIH3T3 fibroblasts, or cortical cells where cultured in the upper Transwell compartment above NSCs from embryonic mouse cerebral cortices	• Only growth factors secreted from endothelial cells promoted fNSC proliferation and self-renewal	127
	• After removal of endothelial cells, fNSCs had an increased propensity for neurogenesis	
Coronal sections of the SVZ of 3‐day‐old SD rats were cultured in the upper Transwell compartment above a monolayer of bEnd.3 cells	• NSCs within coronal sections exhibited enhanced proliferation as well as neurogenesis and gliogenesis when co-cultured with BECs	128
	• BEC-derived VEGF promoted NSC proliferation and differentiation, possibly by activating NSC Notch and Pten pathways	
Primary BECs, pericytes, or astrocytes were isolated from dentate gyri and cortices from 4-week-old C57BL/6 mice and cultured in the upper Transwell compartment above NPCs isolated from the hippocampus	• All cell types enhanced NPC survival	129
	• BECs enhanced neuronal differentiation	
	• BECs and astrocytes enhanced NPC proliferation	
Primary BECs and NPCs were isolated embryonic mice and co-cultured in noncontact or contact Transwell culture	• In noncontact Transwell culture, BECs enhanced NPC self-renewal	130
	• In contact culture, BECs enhanced NPC neurogenesis	
NSC were isolated from postnatal-day-1 C57BL6 mice and co-cultured with bEnd.3 cells in noncontact and contact Transwell culture	• In both co-cultures, NSCs increased NO production	62
	• NSC NO enhanced BEC production of VEGF and BDNF	
	• Vascular VEGF promoted endothelial tube formation	
	• Vascular BDNF stimulated additional NSC NO production	
In a Transwell, bEnd.3 cells were exposed to static or flow conditions using a cone-plate viscometer with NPCs isolated from the SVZ of adult Swiss Webster mice cultured 10 *μ*m or 1000 *μ*m below the Transwell membrane	• BECs secretion of pro-neurogenic factors was maximized in the flow condition with NPCs cultured at 10 μm	131
	• The same condition yielded the most proliferation of Type C cells and Type A cells	
Brain microvessel endothelial cells were isolated from the cortices of adult male Sprague Dawley rats and cultured in the upper Transwell compartment above NPCs isolated from embryonic rat brains	• BEC monolayers co-cultured with NPCs exhibited reduced barrier permeability and increased TEER values	132
	• BECs suppressed NPC neurogenesis	
Human iPSC-ECs were cultured in the upper Transwell compartment above several combinations of primary human fetal pericytes, primary human fetal astrocytes, human iPSC-NSCs, and human fNSCs	• Compared to the iPSC-EC monoculture, only the tri-culture (iPSC-ECs, pericytes, and iPSC-NSCs) and quad-culture (iPSC-EC, pericytes, astrocytes, and iPSC-NSCs) conditions significantly enhanced average TEER values	117
	• Quad-culture condition upregulated several BBB genes	

**TABLE III.** Three-dimensional hydrogels.

Experimental design	Major findings	References
NPCs were isolated from the SVZ of post-natal-day-1 transgenic rats and combined with an immortalized BEC line within poly(ethylene glycol)/poly(L-lysine) hydrogels. Hydrogels were implanted into 8–10-week-old Sprague–Dawley rats	• After six weeks, BECs co-cultured with NPCs produced tubular structures with significantly greater densities than BECs cultured in hydrogels alone	62, 140
	• Blood flow in new microvessels was observed two weeks after hydrogel implantation	
Human NSCs/NPCs were isolated from the cerebral cortices of human fetuses and combined with human ECFC-ECs isolated from cord blood within hydrogels composed of salmon fibrin with interpenetrating networks of hyaluronic acid and laminin	• Compared to fibrin hydrogels, combination scaffolds enhanced NSC/NPC proliferation and differentiation into astrocytes and neurons	123
	• When cocultured with NSCs/NPCs, ECFC-ECs showed enhanced microvessel formation	

**TABLE IV.** Spheroids.

Experimental design	Major findings	References
NSCs were isolated from human fetuses and molded into spheroids with bEnd.3 cells with an array of 500 *μ*m-diameter microwells	• Compared to NSC-only neurospheres, co-spheroids demonstrated enhanced expression of self-renewal and adhesion markers	143
	• Co-spheroids exhibited increased markers for gliogenesis and decreased markers for neurogenesis, possibly through the Notch signaling pathway	
NSCs isolated from adult mouse brain and endothelial cells harvested from the bovine carotid artery were seeded on chitosan-based substrates and readily formed co-spheroids	• Compared NSC neurospheres, co-spheroids exhibited no significant change in self-renewal marker expression	124
	• Co-spheroids exhibited enhanced neuron and astrocyte differentiation markers	
	• When cultured in gelatin-based hydrogels, co-spheroids expressed angiogenic markers and extended vascular tube-like structures	
Spheroids were made of either iPSC-NPCs or iPSC-ECs generated from human iPSK3 cells. These spheroids were merged together with the support of MSCs isolated from the human bone marrow	• Combination spheroids expressed elevated levels of self-renewal markers, ECM proteinases, and BBB genes	116
	• MSC migration within the combination spheroids was found to be CXCR4-dependent	

**TABLE V.** Microfluidic devices.

Experimental design	Major findings	References
NSCs isolated from mouse embryos were resuspended in collagen I and injected into the hydrogel channel of MFDs. bEnd.3 cells were seeded on the sides of the fluidic channels to form vascular channels	• Compared to NSCs alone, NSCs cultured with BEC-lined vascular channels showed enhanced self-renewal, possibly through a vascular-derived PEDF-dependent mechanism	148
	• NSCs in close proximity to the vascular channels demonstrated enhanced astrocyte differentiation	
In MFDs with a central hydrogel channel, H9-derived human NSCs were seeded in one fluidic channel and human BECs were seeded in the opposite channel with MSCs isolated from human bone marrow	• Hydrogels composed of fibrin and Matrigel were able to induce NSC neurogenesis and BEC angiogenesis	149
Human NSC-MNs were aggregated into spheroids and suspend with HUVECs in the central channel of MFDs designed to induce microvascular network formation	• NSC-MN spheroids exhibited spontaneous neuronal activity with microvascular networks, likely due to endothelial-derived BDNF and Notch pathway activation	150
	• Spontaneous neuronal activity was enhanced when microvascular networks were perfused	
MFDs were composed of three connected compartments; two BBB compartments seeded with primary cortical BECs, pericytes, and astrocytes and one brain parenchymal compartment seeded with human hippocampus NSC-derived neural cells	• Untargeted metabolic analysis was performed for each compartment	151
	• Metabolic coupling between the BBB and neuronal cells was identified	
	• Methamphetamine increased BBB permeability	
Human iPSC-derived spNPCs and iBMECs were seeded in neural and vascular channels, respectively, separated by a porous membrane	• iBMECs and spNPCs expressed tight junction proteins and mature neuron markers, respectively	152
	• spNPC-derived neurons cultured with iBMECs in the MFD had the highest expression of neurogenesis gene pathways	
Human patient-specific iBMECs and iPSC-derived NPCs were seeded in vascular and brain channels, respectively. The effects of using iBMECs derived from patients with HD and MCT8 deficiency were observed	• Laminar flow promoted BBB function for iBMECs derived from healthy patients	153
	• iBMECs derived from HD patients had increased dextran permeability	
	• iBMECs derived from patients with MCT8 deficiency had decreased permeability to triiodothyronine	
	• iBMECs decreased NPC-derived neural cell toxicity when the vascular channel was perfused with human whole blood	
ReNcell VM human NPCs were made to express fAD mutations (ReN-AD cells) and suspended in hydrogel channels separated from hCMEC/D3 cell-coated fluidic channels	• ReN-AD cells created an accumulation of amyloid plaque which decreased BEC tight junction protein expression and increased dextran permeability	154
	• Etodolac and beclomethasone decreased permeability and dead ReN-AD cell count	
Human ESC-derived endothelial cells, NPCs, and microglia were combined with primary human pericytes within MFDs to recreate the developing human PNVP	• Model replicated human embryonic SVZ composed of radial glia and differentiated neural cells	155
	• BDNF and differential metabolite secretion increased from the vascular plexus and neuronal layer	
	• Several developmental toxicants reduced angiogenesis, vasculogenesis, and VEGF-A secretion	

### A. Cell sources for *in vitro* models

The generation of viable, homogeneous populations of NSCs/NPCs is essential for the continued efficacy of *in vitro* NVN models. This standard will ensure reproducible results and generate data translatable to clinical settings. A distinct advantage of using animal-derived cells is the relative ease of acquiring primary populations from different regions of the brain. Primary NSCs/NPCs isolated from embryonic and adult mouse brains possess the ability to proliferate and differentiate *ex vivo.*^2,110^ Fetal NSCs/NPCs can be derived from the ganglionic eminence^111^ and the cortical anlage;^111,112^ Adult NSCs/NPCs can be isolated from the SVZ^37,77^ and SGZ.^113^ Despite the advantage of selecting NSC/NPC origin, *ex vivo* studies can be laborious and result in heterogeneous cell populations.^113^ Fortunately, human and animal neural induction protocols have been implemented to generate populations of NSCs/NPCs from both embryonic stem cells (ESCs)^58,114^ and induced pluripotent stem cells (iPSCs).^115–117^ Oh and colleagues found the transcriptome profile of human ESC-derived NSCs to be analogous to that of ReNcell CX cells, a well-characterized immortalized human NPC line.^114^ Furthermore, D'Aiuto and colleagues developed a cost-effective method to produce of large populations of human iPSC-derived NSCs from readily available somatic cells.^118^ These characteristics of induced NSCs/NPCs make them ideal replacements for primary and immortalized NSCs/NPCs in future human studies. In general, NSCs/NPCs procured using any protocol can be expanded as 2D monolayers or 3D neurospheres while maintaining stem cell characteristics for several generations in a culture medium containing EGF and bFGF.^2,71,114,118^

Endothelial cells are major orchestrators of NSC/NPC fate and essential components for modeling the NVN. Like neural cells, BECs from both rodent and human sources are commonly used. For mouse studies, bEnd.3 cells are an immortalized cell line routinely used for BBB studies due their low barrier permeability and high expression of tight junction proteins.^58,106,119^ Human adult cerebral microvascular endothelial (hCMEC/D3) cells are implemented in human BBB studies for similar reasons.^111,112,120^ Although primary human BEC lines are available commercially, first generation primary mouse BECs are routinely obtained directly from different regions of the brain.^77,121^ Like NSCs/NPCs, human ESCs and iPSCs can generate brain-specific endothelial cells that share characteristics with bona fide BECs.^116,117,122^ As an example, trans-endothelial electrical resistance (TEER), an indication of BBB integrity and tight junction formation, was observed to be significantly higher with iPSC-derived BECs compared to hCMEC/D3 cells.^122^ These findings, in addition to the high-volume production of derived BECs, reduce the need for primary and immortalized BEC lines in future human studies. Given the similarities of endothelial cell genomes and secretomes, several *in vitro* studies have investigated the interaction between NSCs/NPCs and nonbrain endothelial cells, including HUVECs,^76^ dermal microvascular endothelial cells,^121^ human endothelial colony-forming cell-derived endothelial cells (ECFC-ECs),^123^ and even bovine carotid artery endothelial cells.^124^ Regardless of the origin, endothelial cells will provide paracrine and juxtacrine signals that influence NSC/NPC behavior in *in vitro* models.

### B. Two-dimensional cell cultures

Most biological research has been performed using conventional cell culture on 2D substrates. Although 2D cell culture does not mimic physiological conditions, the simplicity and cost of 2D systems allow for standardized, high-throughput research. In 2D culture, gain-of-function and loss-of-function experiments can be performed with minimal confounding variables. Conclusions from 2D studies can then be extrapolated to design hypothesis-based experiments in more complex systems. For these reasons, many co-cultures of endothelial cells and NSCs/NPCs on 2D substrates have been used to study their heterotypic cellular interactions [Fig. 2(b)].

To study juxtacrine signaling, different cell types can be cultured in direct contact with each other. Tung and colleagues observed enhanced bEnd.3 cell cytokine and chemokine expression when ESC-derived NSCs/NPCs were co-cultured directly on top of them in cell culture dishes.^58^ As previously mentioned, Ottone and colleagues found that culturing mouse SVZ NSCs/NPCs on a monolayer of bEnd.3 cells induced NSC quiescence and stemness, confirming the importance of NSC contact with brain blood vessels in the NVN.^37^ To show the reciprocal interactions between the BECs and NSCs, Chou and colleagues co-cultured human fNSCs on a monolayer of hCMEC/D3 cells and induced the formation of endothelial cell tubular networks.^111^ These vasculature-like structures expressed tight junction markers and generated basement membranes similar to those of physiological blood vessels. Besides, NSCs cultured with tubular networks experienced enhanced neurogenesis compared to NSCs alone.^111^ A follow-up study revealed that the biological origin of NSCs is also a determinant of endothelial cell morphogenesis. Human fNSCs from the ganglionic eminence induced hCMEC/D3 cell tube formation four times as efficiently as fNSCs from the cortical anlage.^112^ Long-term co-culture with hCMEC/D3 cells decreased the NSC expression of proliferative (Ki67) and stem cell (Sox2 and Nestin) markers and increased differentiation markers (GFAP, MAP2, and GalC). Additionally, pharmacological blocking of VEGF-R2 and laminin-binding integrin subunit α6 inhibited tubular network formation, identifying the protein mechanisms through which NSCs induced endothelial morphogenesis.^112^

Endothelial cells intrinsically form tubular networks when plated on Matrigel-coated 2D substrates.^125^ Indeed, Vissapragada and colleagues observed the tube formation of mouse periventricular embryonic BECs (PVECs), adult BECs, and dermal microvascular endothelial cells in Matrigel-coated wells.^121^ However, only PVECs exhibited increased tube length when co-cultured with mouse NPCs.^121^ These results highlight the influence of endothelial cell origin and age on the responsiveness to NPC signals and provide some insight into the temporal signaling mechanisms of angiogenesis. Even in the presence of oxygen/glucose deprivation (OGD), mouse embryonic NSCs/NPCs cultured directly on bEnd.3 cells promoted endothelial cell morphogenesis, revealing the protective role of NSCs/NPCs.^106^ Tubular network formation was stimulated by neural HIF-1α and VEGF; both of which were upregulated in NSCs/NPCs when co-cultured with BECs under OGD conditions.^106^ Further insight regarding HIF-1α and VEGF signaling pathways will be necessary to understand the therapeutic influence of NSCs/NPCs on neo-vascularization in the brain. Despite the simplicity of these direct cell contact 2D systems, they are highly valuable for investigations regarding cell-specific molecular mechanisms of NSC neurogenesis and endothelial cell morphogenesis.

The effects of cell-secreted diffusible signals can be assessed in 2D systems by culturing target cells in source cell-conditioned medium. Crouch and colleagues cultured mouse NSCs/NPCs in medium conditioned with BECs and pericytes isolated from neurogenic (SVZ) and non-neurogenic (cortex) regions of the adult mouse brain.^77^ Although vascular cell-derived soluble factors from both brain regions promoted NSC/NPC proliferation and neurogenesis, cortical vascular cells had the most influential effect, highlighting the intrinsic neuroprotective ability of BECs outside of the NVN.^77^ Notwithstanding 2D cell culture constraints, Dumont and colleagues demonstrated the influence of flow on endothelial cell-derived soluble factors and NPC fate.^126^ Briefly, a cone-plate viscometer exposed bEnd.3 cells to a shear stress of 0 (static) or 10 dynes/cm^2^ (flow) and adult mouse NPCs were cultured with conditioned medium from both regimes. Conditioned medium from static and flow conditions promoted oligodendrocyte and neuron differentiation, respectively. Under flow conditions, BECs demonstrated enhanced secretion of EGF and bFGF, in addition to HSPGs which likely increased growth factor bioactivity similar to the way that HSPG-rich fractones have *in vivo.*^126^ These data suggest that the release of BEC-derived soluble factors is flow-dependent and emphasize the capacity of *in vitro* models to replicate physiological conditions.

Transwell systems allow soluble factor diffusion and are critical for investigating paracrine signaling between BECs and NSCs/NPCs. Upper and lower compartments separated by a microporous membrane enable contact and noncontact studies [Fig. 2(b)]. Transwell systems allow soluble factor diffusion and are critical for investigating paracrine signaling between BECs and NSCs/NPCs. Shen and colleagues conducted seminal work on the effect of diffusible signals from different feeder cells on mouse NSCs in a noncontact Transwell system.^127^ Only growth factors secreted from endothelial cells promoted NSC self-renewal, demonstrated by increased nestin expression and Hes1 activation. After the Transwell insert with endothelial cells was removed, NSCs had an increased propensity to differentiate to neurons (TuJ1+ and MAP2+), inferring that endothelial cells prime NSCs for neurogenesis.^127^ Similarly, Sun and colleagues observed that NSCs in rodent brain slices exhibited enhanced proliferation as well as neurogenesis and gliogenesis when co-cultured with BECs in Transwell systems.^128^ RNA interference of vascular VEGF diminished these observations and identified the involvement of the Notch and Pten signaling pathways in NSC proliferation and differentiation.^128^ Transwell systems have also been implemented to compare the influence of soluble factors from different NVN cell sources. Ehret and colleagues isolated BECs, pericytes, and astrocytes from mouse brains and established a noncontact co-culture with SGZ NPCs.^129^ All cell types enhanced NPC survival, but only BECs increased neuronal differentiation. In addition, both BECs and astrocytes improved NPC proliferation, revealing the glial cell contributions in the NVN.^129^ Although these data highlight the predominant influence of endothelial cell-derived paracrine signals in the NVN, they also reveal the impact of nonvascular cell types on NSC/NPC behavior.

Transwell systems can be used to identify the cellular mechanisms involved in contact and noncontact cell culture. Mouse embryonic NPCs maintained a progenitor phenotype with high nestin expression when cultured in noncontact conditions with embryonic BECs.^130^ Interestingly, co-culturing the same cells in direct contact resulted in the preferential differentiation of NPCs to neurons (MAP2+).^130^ These results highlight the distinctive effect of paracrine and juxtacrine signaling mechanisms involved in NSC self-renewal and neurogenesis. In both direct contact and noncontact culture with BECs, postnatal mouse NSCs increased NO production which enhanced BEC expression of VEGF and BDNF.^62^ Vascular VEGF induced endothelial tube formation and BDNF stimulated further NSC NO production, creating a positive feedback loop.^62^ The understanding of the reciprocal modulation between NSCs and BECs will be critical for developing cell therapies using both cell types.

Expanding upon their previous work, Dumont and colleagues investigated the effect of the hemodynamic state and spatial arrangement of BECs on NPC fate by integrating a Transwell insert with a viscometer.^131^ In the upper compartment, bEnd.3 cell monolayers were subjected to static or flow conditions with adult mouse NPCs cultured 10 *μ*m or 1000 *μ*m below the Transwell membrane. The flow condition with NPCs cultured at 10 *μ*m yielded the greatest release of BEC-derived pro-neurogenic factors as well as the largest populations of Type C cells (Oligo2+ or Mash1+) and Type A cells (PSA-NCAM+).^131^ These results emphasize the importance of shear stress-induced growth factor secretion and how their effect on NPCs is influenced by proximity. Since the behavior of NSCs/NPCs in the NVN is largely governed by their spatial relationship to blood vessels, further investigations into the cellular mechanisms of this interrelation need to be performed.

Transwell systems have also been used to investigate the influence of NSCs/NPCs on the function of BECs. The TEER values of endothelial cell monolayers cultured in the upper compartment of Transwell systems can be easily measured using a voltohmmeter, as illustrated in Fig. 2(b). Weidenfeller and colleagues reported that BEC monolayers co-cultured with NPCs exhibited reduced barrier permeability and increased TEER values compared to BECs alone.^132^ In a similar Transwell system, Appelt-Menzel and colleagues reported the effects of several human cell types on the barrier function of human iPSC-derived endothelial cells (iPSC-ECs).^117^ However, neither iPSC-NSCs nor fNSCs significantly increased iPSC-EC TEER values. Only the tri-culture (iPSC-ECs, pericytes, and iPSC-NSCs) and quad-culture (iPSC-EC, pericytes, astrocytes, and iPSC-NSCs) conditions enhanced TEER values compared to the iPSC-EC monoculture. In the quad-culture condition, iPSC-ECs upregulated the expression of membrane transporters and occludin, all of which are characteristics of the BBB.^117^ Taken together, these data indicate that several cell types, not just NSCs/NPCs, contribute to the integrity of the BBB in the NVN. The contrasting TEER results between Weidenfeller and Appelt-Menzel could be attributed to differences in NSC/NPC and endothelial cell origin and secretomes. Future studies could investigate the effect of NSC source and lineage on BEC function.

Collectively, 2D *in vitro* models have been instrumental to the understanding of the cellular interaction within the NVN. Conventional cell culture offers advantages such as simple culture protocols and easy to interrupt results. However, cells cultured on stiff 2D substrates lack physiological relevancy. Tissue culture dishes induce apical-basal cell polarity and cannot generate soluble factor gradients. Furthermore, the lack of proper cell–cell and cell–ECM interactions results in cells adopting morphologies and functions distinct from those observed *in vivo.*^133,134^ Therefore, to properly recapitulate the crosstalk between BEC and NSCs/NPCs *in vitro*, a 3D microenvironment must be created.

Another conspicuous feature for most of the studies mentioned in this section is the use of rodent-derived cells. This can be attributed to the fact that the majority of these studies were performed nearly a decade ago. Stem cell-derived or immortalized human cell lines were not as available or well-characterized at the time. Moreover, the use of rodent-derived cells cannot be classified exclusively as a negative attribute. Rodents have been the most commonly used an animal model for biological research for many years and *in vitro* experimentation using rodent cells was certainly paramount for the development of ethical, hypothesis-driven studies.^29^ However, moving forward, it goes without saying that the use of human-derived cells will be preferred when developing *in vitro* models of the NVN.

### C. Three-dimensional hydrogels

Unlike 2D substrate-based cell culture, 3D cell cultures utilized hydrogels that suspend cells in a matrix and microenvironment mechanically similar to what they would experience *in vivo*. Hydrogels facilitate spatial gradients as well as omnidirectional cell spreading and migration, as illustrated in Fig. 2(c).^134^ In addition, the physical qualities of hydrogels (composition, stiffness, porosity, charge, and ECM ligand density) can be attuned to optimize cell morphology and function.^135^ In 3D matrices, endothelial cells undergo natural morphogenic processes to produce microvessels with hollow lumen and vessel polarity.^136^ Hydrogels have also been implemented to promote NSC/NPC self-renewal^137^ and neurogenesis.^138^ Despite these advantages, there is a paucity of hydrogel studies investigating the interaction of NSCs and BECs. Ford and colleagues first demonstrated that mouse BECs co-cultured with NPCs within a macroporous poly(ethylene glycol)/poly(L-lysine) hydrogel showed enhanced microvessel formation after subcutaneous implantation into mice compared to hydrogels containing only BECs.^139^ Microvascular networks were stable up to six weeks and anastomosed with host blood vessels, indicating that NPCs support stable vascular network formation.^139,140^ These results in a living organism are encouraging for future clinical applications, however, the previous studies were performed in mice. To study human NSCs/NPCs, Arulmoli and colleagues created a hydrogel scaffold with similar physical properties to brain ECM by combining salmon fibrin with interpenetrating networks of hyaluronic acid and laminin.^123^ Due to the fibrinogen-binding integrins (αVβ1 and α5β1) and laminin-binding integrins (α3β1, α6β1, α7β1), NSCs/NPCs exhibited increased proliferation and differentiation into astrocytes and neurons in combination scaffolds. When cocultured with NSCs/NPCs, human ECFC-ECs showed enhanced microvessel formation, demonstrating the combination scaffold's ability to support vascularization highlighting the supporting role of NSCs/NPCs.^123^ These results show that 3D hydrogel systems can be used to mimic the cell–cell and cell–ECM interactions of NSCs/NPCs and BECs that are impossible to recreate in conventional cell culture. As such, endothelial and neural cells are more likely to adopt their native morphologies and functions. Notwithstanding the advantages, the added complexity of hydrogel studies means that experimental reproducibility and data interpretation may be more difficult than in conventional cell culture.^133^ Furthermore, the use of hydrogels requires additional costs and skills needed for experimental protocols. However, to ensure the success of cell therapies in clinical trials, *in vitro* models that recapitulate the 3D microenvironment of the NVN will be needed to predict how NSCs/NPCs and BECs behave *in vivo*. It is conceivable that both cell types will be introduced simultaneously into human patients with a supportive ECM, similar to the experimental design implemented by Ford and colleagues.^139,140^

### D. Spheroids

One common method to study NSC biology is to aggregate NSCs/NPCs into neurospheres. Neurospheres are high-throughput models used to understand the cell–cell and cell–ECM mechanisms that govern NSC/NPC fate.^141^ Like neurospheres, spheroids are amalgamations of one or several cell types combined with or without supporting ECM proteins. These clusters are considered superior to conventional cell culture because they situate cells in a microenvironment to induce natural cell characteristics.^142^ This technique has been implemented to study the interactions between endothelial cells and NSCs/NPCs, as illustrated by Fig. 2(d). Yang and colleagues induced the self-assembly of co-spheroids containing human fNSCs with bEnd.3 cells in microwells.^143^ Co-spheroids had enhanced expression in self-renewal markers (Nestin, Hes1, and Hes5) and adhesion molecules (E-cadherin and N-cadherin) compared to fNSC neurospheres. In addition, co-spheroids exhibited increased markers for gliogenesis (GFAP) and decreased markers for neurogenesis (TuJ1 and MAP2), likely due to endothelial cell-induced Notch signaling which directs NSCs toward glial differentiation.^143^ Similarly, Han and colleagues reported that adult mouse NSCs and bovine carotid artery endothelial cells cultured on chitosan-hyaluronan substrates readily formed co-spheroids.^124^ Unlike the previous study, co-spheroids showed an increase in both neurogenesis and gliogenesis compared to NSC-only neurospheres. The source of the discrepancy between the two studies is unclear but likely due to the use of different cell types.^124,143^ In addition, co-spheroids exhibited the growth of capillary-like structures and enhanced expression of angiogenesis markers when cultured in bFGF-bound gelatin-based hydrogels.^124^ These data demonstrated the angiogenic potential of these co-spheroids. Given the combination of endothelial cells and NSCs/NPCs for prospective neurodegenerative therapies, these results instill confidence in spheroid-based neo-vascularization and neo-neurogenesis in human patients. Hybrid spheroids were developed by Song and colleagues by promoting the fusion of human iPSC-NPC spheroids and iPSC-EC spheroids with support from mesenchymal stem cells (MSCs).^116^ Hybrid spheroids expressed elevated levels of self-renewal markers (Notch1), ECM proteinases (MMP2 and MMP3), and BBB genes (GLUT-1 and ZO-1). In addition, a CXCR4 antagonist was used to demonstrate that MSC migration during spheroid fusion was CXCR4-dependent, similar to Type B, Type C, and Type A cell chemotaxis in the SVZ.^68,116^ These data show the prominent roles of both cell–cell and cell–ECM interactions during hybrid spheroid formation and infer that similar interactions are present in the native NVN. In summary, although spheroids are excellent models of heterotypic cell interactions, few studies use spheroids composed of NSCs/NPCs and endothelial cells. Spheroids can recreate tissue cytoarchitecture and more accurately recreate cell–cell interplay. Furthermore, the ease of production makes spheroid culture ideal for high-throughput mechanistic and pharmacological studies for diseases, such as neurodegenerative disorders.^142^ However, like neurosphere generation, sensitivity to variations in generation procedures can result in heterogeneous cell populations between different studies.^142,144^ Therefore, standardization of cell sources and quality assurance tests must be implemented to spheroid studies involving BECs and NSCs/NPCs.

### E. Microfluidic devices

MFDs are experimental platforms that manipulate minute volumes of fluid, resulting in the cost-effective use of culture reagents. Although the soft-lithography fabrication process of many MFDs requires additional costs and experimental skills, it also allows MFDs to be customized to meet the specific needs of an experiment. MFDs can precisely control spatiotemporal experimental parameters, as illustrated in Fig. 2(e), making them exemplary systems to mimic biochemical microenvironments. While MFDs have previously been developed to model the BBB^145,146^ and study neural tissue engineering,^147^ there are fewer microfluidic models that specifically investigate the cellular relationships of the NVN. Shin and colleagues were one of the first to develop a MFD that studied the interaction of mouse embryonic NSCs and bEnd.3 cells.^148^ NSCs cultured between BECs-coated vascular channels had increased proliferation and self-renewal marker expression (Nestin, Hes1, and Hes5). Compared to 2D and 3D Transwell controls, NSC nestin upregulation in the presence of BECs was dramatically enhanced in MFDs, highlighting the benefit of using MFDs to study cell–cell interactions.^148^ Interestingly, the vascular channels increased differentiation to astrocytes (GFAP+) and oligodendrocytes (Oligo2+) but reduced differentiation to neurons (TuJ1+). This was seemingly in contrast to the neurotrophic effect of BECs reported in previous studies.^127^ However, NSC spatial analysis revealed that NSCs closer to the vascular channels had enhanced astrocyte differentiation.^148^ This aligned with the contact-mediated gliogenesis previously mentioned and explained the preferential glial differentiation.^143^ Moreover, vascular PEDF knockdown decreased NSC self-renewal marker expression which corroborated with observations reported in the mouse SVZ.^67,148^ While these results evidenced the use of MFD models to confirm discoveries found in animal models, this study used murine cells. To elucidate the cellular mechanisms of the human NVN, primary or stem cell-derived human cells will be needed.

As previously stated, Louissaint and colleagues demonstrated that angiogenesis and neurogenesis were coupled processes.^60^ Developing neurovascular tissue in MFD models will lead to a better understanding of how the two processes are connected. Uwamori and colleagues created a MFD that promoted human NSC neurogenesis and microvascular network formation in a 3D fibrin-Matrigel hydrogel.^149^ While the authors speculated that this platform could be used to investigate the cellular mechanisms of neurogenesis and angiogenesis in a 3D microenvironment, no such analysis was done. Osaki and colleagues investigated neurovascular tissue more extensively by culturing NSC-derived motor neuron (NSC-MN) spheroids in a MFD designed to develop perfused microvascular networks of HUVECs.^150^ When cultured with microvascular networks, the NSC-MN spheroids exhibited enhanced neurite extension and spontaneous calcium oscillation. This was likely due to HUVEC BDNF secretion and increased endothelial-neural contact-induced Notch pathway activation. The authors also observed that perfused microvascular networks caused enhanced spontaneous neuronal activity in MSC-MN spheroids when compared to static conditions.^150^ Luminal flow is a critical feature of the NVN that is absent in most 2D models. These data demonstrate the importance of vascular perfusion for neuronal activity and accentuate the superiority of using MFDs to determine cellular mechanisms of neurogenesis.

When identifying the effects of BEC-derived signals in the native NVN, it is difficult to conclusively determine the signal source and contribution due to the influence of other cell types.^16^ However, MFDs have been used to decouple the cellular interactions of neurovascular tissues. Maoz and colleagues created a linked “organ-on-a-chip” composed of three connected microfluidic compartments to study the brain parenchymal region as well as the influx and efflux across the BBB.^151^ The two BBB compartments were composed of a monolayer of primary human BECs separated by a membrane from primary human pericytes and astrocytes. The brain compartment contained a mixed population of neuronal and glial cells differentiated from human hippocampus-derived NSCs. The authors performed untargeted metabolic analysis for each compartment, identified previously unknown metabolic coupling between the BBB and neuronal cells, and demonstrated the reversible effect of methamphetamine on BBB permeability.^151^ The authors claimed that their organ-on-a-chip mimicked the physiological functions of the neurovascular unit more efficiently than static cultures in Transwells. It is currently unknown if the metabolic products of BECs directly affect NSC/NPC metabolism. By applying Maoz's concept of segmenting functional units of the brain, future studies could identify previously unknown metabolic coupling within the NVN.

The primary role of the mammalian NVN is to maintain neurogenesis into adulthood. MFDs have been successfully implemented to study this transition by observing the differentiation from NPCs to functional neurons. Sances and colleagues developed a MFD with two fluidic channels separated by a porous membrane; one channel lined with human iPSC-derived brain microvascular endothelial cells (iBMECs) and one seeded with iPSC-derived spinal NPCs (spNPCs).^152^ The iBMECs formed a functional endothelial monolayer with tight junction proteins (Occludin and ZO-1) and the spNPCs expressed mature neuron markers (TuJ1, MAP2, and synaptophysin). Analysis of spontaneous calcium signaling in spNPC-derived neurons revealed that the highest neuronal activity was seen in MFDs with iBMECs, compared to MFDs without iBMECs as well as in 96-well plates. RNA sequencing showed that spNPCs cultured with iBMECs in MFDs had a higher expression of neural differentiation and neurogenesis gene pathways.^152^ These data not only emphasize the role of BECs in neurogenesis but, again, accentuate the distinction between conventional and MFD cell culture when mimicking physiological neurogenesis.

Using a similar technique, Vatine and colleagues seeded patient-specific iBMECs and iPSC-derived NPCs in the previously mentioned MFD.^153^ The brain compartment contained NPCs (Nestin+), astrocytes (GFAP+), and neurons (TuJ1+ and MAP2+) which demonstrated spontaneous neuronal calcium activity. Unlike the previous study, laminar flow was introduced to the vascular channel to create shear stress across the endothelium which has been shown to enhance BBB characteristics in previous MFD models.^145^ Indeed, for iBMECs derived from healthy patients, laminar flow upregulated tight junction related genes and produced TEER and dextran permeability values that indicated barrier formation.^153^ When using iBMECs derived from patients with Huntington's disease (HD), dextran permeability increased, indicating compromised BBB function. In addition, iBMECs derived from patients with monocarboxylate transporter 8 (MCT8) deficiency showed a decrease in the permeability of triiodothyronine which requires functional MCT8 to cross the BBB. The authors then perfused whole human blood through the vascular channel and showed that the presence of the iBMEC monolayer decreased neural cell toxicity. Using whole blood, decreased triiodothyronine permeability was observed again when using iBMECs derived from patients with MCT8 deficiency.^153^ With the distinct use of whole blood and iPSC-derived cells from individual humans, the authors demonstrated the application of MFDs to study patient-specific neurodegenerative diseases. Similar designs could be implemented to investigate the effects of neurological disorders on NVN functions, such as NSC self-renewal and neurogenesis. To study the effects of Alzheimer's disease on BBB dysfunction, Shin and colleagues expressed familial Alzheimer's disease (fAD) mutations in ReNcell VM human NPCs (ReN-AD cells) which induced extracellular deposition of amyloid plaques.^154^ Their MFD design contained suspended ReN-AD cells and hCMEC/D3 cell-coated fluidic channels separated by a collagen matrix. Compared to wild-type ReN cells, ReN-AD cells caused an accumulation of amyloid plaque at the vascular endothelium which decreased the expression of several tight junction proteins and increased BBB permeability. The introduction of several AD drugs to the vascular channel decreased BBB permeability and reduced the number of dead ReN-AD cells.^154^ These data demonstrate the efficacy of MFDs to both simulate neurodegenerative disease pathology and assess new pharmacological compounds for treatment.

The versatility of MFD fabrication allows researchers to create models that mimic the cytoarchitecture of biological tissues better than conventional cell culture while also providing the ability to observe cells in real-time which is not possible in animal models. Kaushik and colleagues integrated a 96-well plate with microfluidics to reconstitute the human embryonic perineural vascular plexus (PNVP) using human ESC-derived endothelial cells, NPCs, and other supporting cells.^155^ The model contained a vascular plexus region overlaid by a neuronal layer complete with simulated ventricle and SVZ composed of differentiated neuronal cells (TuJ1+ and GFAP+) and radial glia (Nestin+ and Notch2NL+). Over a 21-day period, BDNF and differential metabolite secretion increased from both the vascular plexus and the neuronal layer, indicating proper PNVP development. The authors observed reduced angiogenesis, vascular network formation, and VEGF-A secretion when several developmental toxicants were introduced demonstrated the efficacy of this model for studying toxicity in the embryonic human PNVP.^155^ The extent to which the human PNVP was reproduced *in vitro* is encouraging evidence that microfluidic models will eventually remove the need for animal models when testing biological hazards.

Results from recent MFD models are encouraging for the future of using these systems to explore the specific cellular mechanisms of the human NVN. Newer designs, using exclusively human-derived cells, have successfully recreated the physiological features of the NVN, allowing cells to adopt their native morphology and function.^150,155^ This capability, in conjunction with the ability to isolate specific cell populations and identify transcriptomic signatures, make MFDs particularly efficient for studying the cellular interactions between human BECs and NSCs/NPCs. Furthermore, the use of human iPSC-derived cells in MFDs has increased the capacity to accurately model the progression of patient-specific neurological disorders.^153,154^ However, several studies discussed in this section have vascularized channels or networks without luminal flow.^148,152,154,155^ Given the importance of endothelial shear stress for BBB formation, going forward, microfluidic models should consider this a paramount feature.^145^ This is also of significance since MFDs have the capacity to investigate the effect of drugs and blood circulating factors on NSCs/NPCs.

Presently, MFD design variability is the field's largest obstacle toward large scale implementation in clinical trials as there is currently no standardization of data measurements.^145^ BBB functions, such as TEER and permeability, are calculated differently depending of MFD design. In addition, the use of different cell types confounds the results from individual studies. However, the widespread adoption of human stem cell-derived cell sources may mitigate these discrepancies. Finally, with regard to pharmaceutical development, translating drug dose data from MFD to human patients is still a daunting task in the nascent field of quantitative systems pharmacology.^156^ Nonetheless, since overcoming this obstacle requires using pharmacokinetic models to perform metadata analysis, more data acquired from microfluidic models are needed. Despite the challenges, the demand for the shift away from animal models has propelled the advancement of MFDs in biological research. The ability of MFDs to identify heterotypic cell–cell interactions in physiologically relevant systems make them ideal candidates to discover the crosstalk between human NSCs/NPCs and BECs.

## V. CONCLUSIONS AND FUTURE PERSPECTIVES

Blood vessels have been identified as an integral component of the NVN and have significant roles in the regulation of NSC/NPC self-renewal, differentiation, proliferation, and migration.^8,16^ The use of animal models to identify the reciprocal interactions between vascular endothelial cells and NSCs/NPCs is currently essential for the progression of neuroscience. However, the generation of novel *in vitro* systems will be needed to model these interactions for human cells due to the physical limitations of using human subjects. Moreover, human *in vitro* models will be able to generate data with more translatability toward drug and cell therapies for human neurological disorders. Conventional cell cultures allow for direct cellular and molecular analysis that is otherwise difficult to conduct in living tissue. Nonetheless, multiplex *in vitro* models that imitate human physiology are needed to yield data for clinical application.

The advancement of the understanding of NVN biology arises from the interpretation of various *in vitro* models. In general, endothelial cell-derived molecules that are postulated to influence NSC/NPC fate can be identified in 2D cell culture.^37,62,77^ These findings engender hypothesis-based studies using more complex *in vitro* models to confirm the physiological relevancy of the data. The ability of 3D *in vitro* models to mimic NVN cytoarchitecture and biochemistry gives credence to their results. In 2006, Ramírez-Castillejo and colleagues identified PEDF as a mouse SVZ niche factor for NSC self-renewal, but stated that it was released by both endothelial and ependymal cells.^67^ Years later, Shin and colleagues confirmed the relationship specifically between endothelial cell-derived PEDF and NSC self-renewal within a MFD.^148^ While not nearly as complex as animal models, modern *in vitro* systems are beginning to bridge the gap between the two experimental systems. To continue this progression, additional 3D hydrogel, spheroid, and MFD models of the NVN must be produced. As previously discussed, most of the conventional cell culture systems developed around a decade ago used rodent cells. It is encouraging that most *in vitro* systems from the past five years, specifically MFDs, have adopted the use of human-derived cells.

This review mainly focused on *in vitro* systems that only used endothelial cells and NSCs/NPCs. While it is necessary to discover legitimate vascular–neural interactions, future *in vitro* models will need to incorporate additional features of the NVN. This includes introducing more NVN cell types, such as pericytes, astrocytes, and even ependymal cells. In addition, *in vitro* models should utilize NVN cells derived from the SGZ niche since it is less characterized than the SVZ niche. Moreover, implementation of stem cell-derived human cell lines may be necessary to standardize results for future analysis. The ability of certain *in vitro* models to recreate blood vessels with open lumen presents the opportunity to study topics difficult to recreate in conventional cell culture, such as fractones, blood-borne factors, and CNS drug delivery across the BBB.^146,148^ Furthermore, while much is known about the influence of endothelial cells on NSCs/NPCs, less is known about the reciprocal interaction. The success of the co-transplantation of endothelial cells and NSCs/NPCs for neurodegenerative disease treatment will be dependent on this knowledge.^19,20^ In summary, while animal models have been imperative for the current understanding of the NVN, the concerted effort of *in vitro* studies will be necessary to increase what is known about the vascular influence of NSC/NPC fate and improve translatability to clinical applications.

## Data Availability

Data sharing is not applicable to this article as no new data were created or analyzed in this study.
